# Evidence-based design of a patient-specific quality assurance program through retrospective analysis of pretreatment and *in vivo* results

**DOI:** 10.1016/j.phro.2026.101106

**Published:** 2026-10-09

**Authors:** Esther Decabooter, Sebastiaan M.J.J.G. Nijsten

**Affiliations:** a Maastro, Maastricht Radiation Oncology Institute, Maastricht, The Netherlands; b Department of Radiation Oncology, GROW Research Institute for Oncology and Reproduction, Maastricht University Medical Centre+, Maastricht, The Netherlands

**Keywords:** Patient-specific QA, Secondary dose calculation, Log file based PSQA, Electronic Portal Dosimetry, *In vivo* dosimetry, Retrospective analysis

## Abstract

**Background and purpose:**

As radiotherapy techniques become increasingly complex, patient-specific quality assurance (PSQA) must evolve to detect a broad spectrum of potential failure modes across the treatment chain. This study presents a extensive evaluation of a multi-modal PSQA program by assessing its ability to detect clinically relevant failure modes and quantifying its efficacy.

**Materials and Methods:**

A five-year retrospective analysis was conducted of an integrated PSQA program implemented at Maastro, combining secondary dose calculations, log-file based verification, and Electronic Portal Dosimetry (EPD) in both pretreatment and *in vivo* workflows. The efficacy of individual QA methods and the overall program was quantified using positive predictive values (PPVs). PSQA outcomes were linked to potential improvements in treatment planning strategies and delivery techniques.

**Results:**

A total of 18,558 treatment plans were analyzed in the pretreatment workflow and 1817 treatment plans in the *in vivo* workflow. Nearly all high-priority failure modes could be detected except machine output miscalibration. Combining PSQA methods substantially improved PPVs across failure categories. The *in vivo* workflow flagged the most failure modes and additionally gives insight into possible improvements in planning strategies or treatment techniques.

**Conclusions:**

This retrospective analysis demonstrated that a comprehensive framework incorporating independent, purpose-specific PSQA methods is essential to detect a wide range of treatment deviations. Combining complementary methods improves the reliability and interpretability of results. The clinical impact of a PSQA program can be further enhanced by adopting more clinically meaningful evaluation metrics and using the results to guide the optimization of new treatment strategies.

## Highlights

• Retrospective analysis of 18,558 pretreatment and 1817 *in vivo* treatment plans.
• Large-scale analysis revealed strengths and limitations of current frameworks.
• Combination of methods increased positive predictive values.
• Additionally *in vivo* results could evaluate new techniques and planning strategies.
• Use of combined, independent and purpose-specific methods is recommended.

## Graphical abstract

**Figure:**
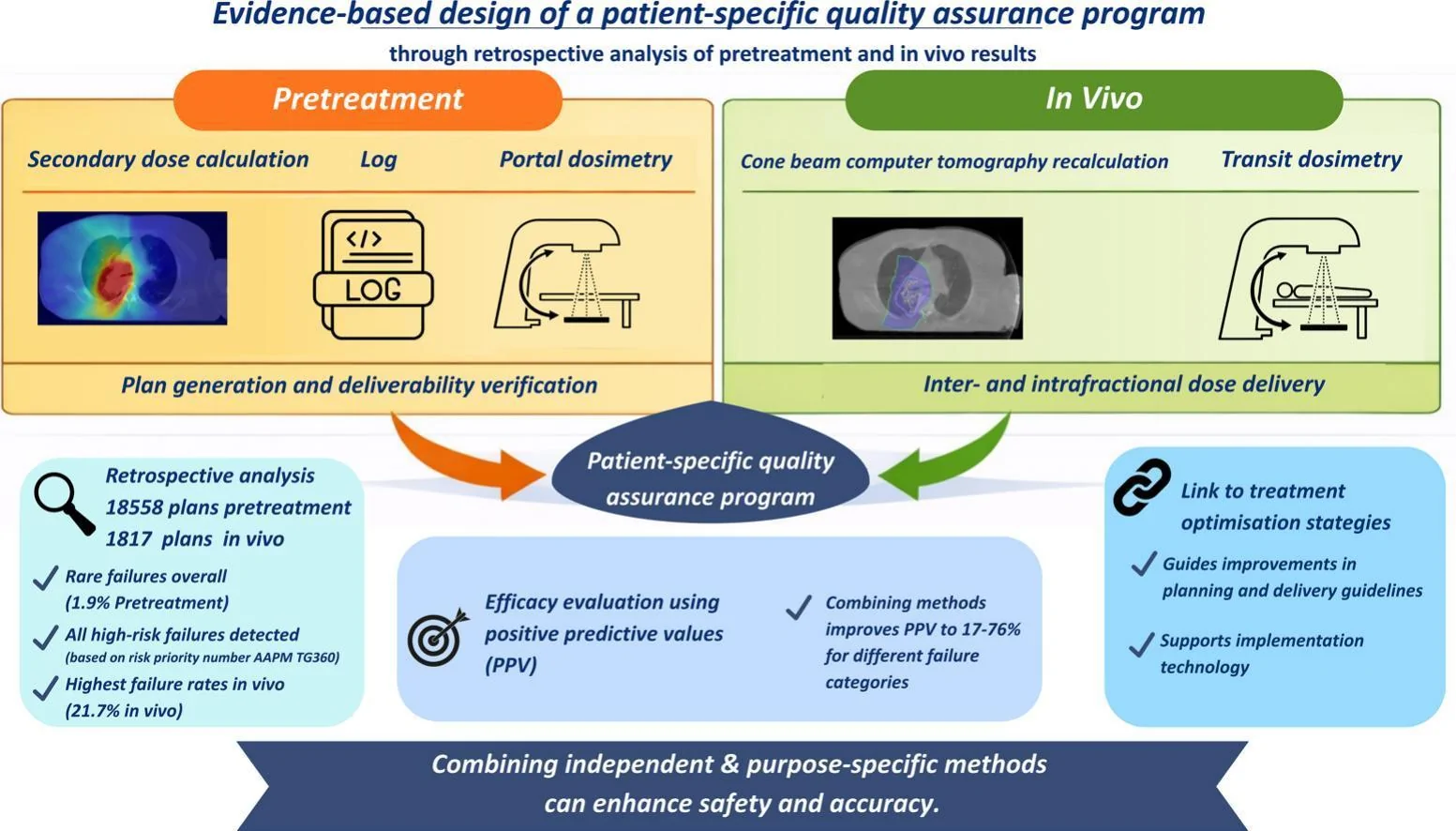

## 1 Introduction

As radiotherapy treatment modalities get increasingly more complex, patient-specific quality assurance (PSQA) becomes more important in ensuring accurate and safe dose delivery. Conventional phantom measurement based PSQA approaches including ionization chamber measurements [1], [2], [3], [4], film measurements [5], [6], [7] or electronic portal imaging device (EPID) measurements [8], [9], [10], [11] have historically formed the basis for verifying treatment plans prior to delivery. However, the introduction of highly modulated techniques including volumetrically modulated arc therapy (VMAT), stereotactic body radiotherapy (SBRT) and adaptive radiotherapy have increased the number and diversity of possible failure modes. These methods alone may not adequately capture the full spectrum of potential failure modes. Advances in cone-beam computed tomography (CBCT) and the availability of on-board EPID have enabled the development of *in vivo* dose verification techniques, providing an additional and complementary layer of quality assurance during treatment delivery [12], [13], [14]. As a result, PSQA is no longer limited to pretreatment plan validation but can also be used to monitor the accuracy and consistency of dose delivery throughout the entire treatment course.

This study presents a retrospective analysis of PSQA data over the last five years. Retrospective analyses of PSQA data remain scarce, with most large-scale efforts concentrating on treatment delivery assessed through a single PSQA modality [15], [16]. This PSQA program integrates multiple PSQA components currently used in clinical practice: secondary dose calculations (SDC) using an independent dose calculation algorithm, dose verification based on machine log files (LOG), and Electronic Portal Dosimetry (EPD) both pretreatment and *in vivo*. These in-house developed PSQA methods are integrated into an automated framework. While PSQA is widely implemented, its ability to consistently detect clinically meaningful errors, its diagnostic value, and its contribution to improving patient outcomes are still under active investigation [17].

The aim of this study was to evaluate an institutional PSQA program through three objectives. First, the current program was assessed whether it identifies clinically relevant failure modes using a large retrospective analysis of PSQA results. Second, the effectiveness of the PSQA program was quantified by determining the positive predictive values (PPVs) of individual PSQA methods and the overall workflow, thereby identifying how reliably detected deviations represent true errors. Third, it was explored how PSQA can improve treatment planning strategies and delivery techniques. Ultimately, this work aimed to provide a critical evaluation of a complete multi-modal PSQA program and evidence-based insights to support the optimization of future PSQA strategies.

## 2 Materials and methods

### 2.1 PSQA program

Fig. 1 illustrates the in-house developed PSQA program containing both a pretreatment plan validation workflow and an *in vivo* treatment delivery verification workflow. All PSQA methods are based on three-dimensional (3D) dose reconstructions derived from either predicted or measured two-dimensional portal dose images (PDIs). The reconstructed dose reconstructions are compared to either the treatment planning dose or SDC using a global 3D gamma analysis. Failed results are reviewed by a medical physicist to determine whether corrective actions or plan adaptations are required.

**Fig. 1:**
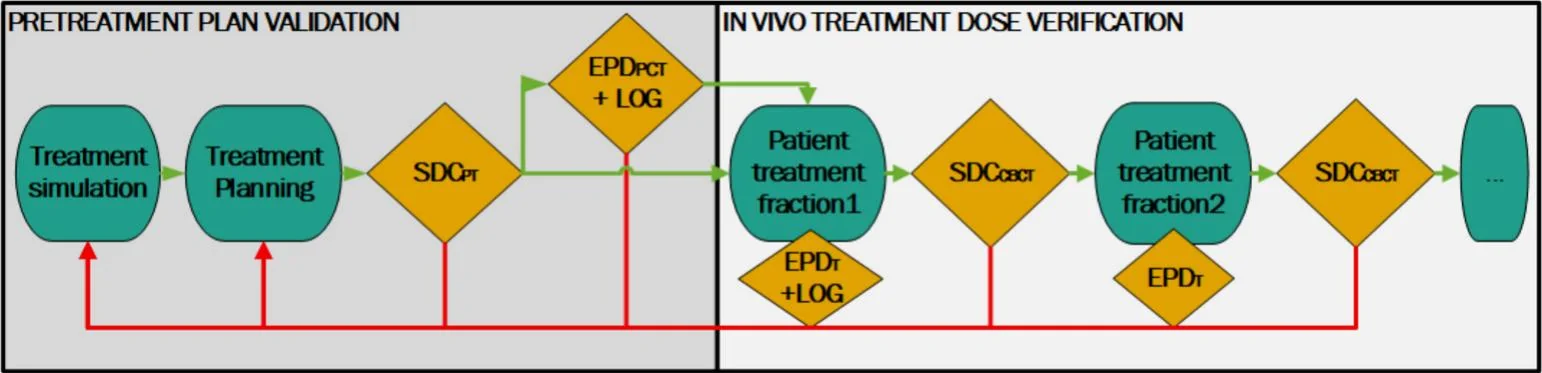
Illustration PSQA program.

Pretreatment plan validation comprises three PSQA methods: a secondary dose calculation on a planning CT (SDC_PCT_), a dose verification based on machine log files (LOG), and EPD using pretreatment acquired portal images (EPD_P_). For all pretreatment methods, the dose is compared to the treatment planning dose. Log file PSQA results are in turn used to identify interrupted deliveries and erroneous EPID acquisitions.

*In vivo* treatment dose verification was performed throughout treatment and consists out of two secondary dose reconstructions based on kV Cone Beam CT scans using 1) the predicted 2D portal dose images based on the original treatment plan (SDC_CBCT_) or 2) the measured transit relative portal dose images (EPD_T_). Each SDC_CBCT_ dose was compared to the SDC_PCT_ dose to minimize dose differences due to 3D dose calculation algorithms. A single fraction with a failed SDC_CBCT_ resulted in the entire plan being marked as failed for this PSQA method. For patients passing SDC_CBCT_s, EPD_T_ measurements were further evaluated. Transit portal dose verification is performed for a selection of patients treated with a curative intent during the first three fractions of the treatment course and then once a week. Additional technical details are provided in the Supplementary Materials A.

### 2.2 Evaluation methods

A retrospective analysis of the pretreatment workflow was performed on 18,558 treatment plans delivered between January 2020 and December 2024 at Maastro (IRB PO0593). Treatment plans with failed secondary dose calculations were excluded from subsequent LOG or EPD_P_ evaluations. Consequently, LOG or EPD_P_ analysis where available for 14,380 plans and 11,885 plans, respectively. The *in vivo* workflow was evaluated between February 2024 and December 2024 and included 13,658 SDC_CBCT_ evaluations from 1817 unique treatment plans. Plans with failed SDC_CBCT_ results were excluded from EPD_T_ evaluation, leaving 1432 plans for analysis. Treatment site, technique, and machine distributions are provided in Fig. 2. All treatment plans were generated using Eclipse treatment planning system (TPS) using either the Acuros (v15.5.11 or v16.1.0) or AAA (v15.5.11 or v16.1.0) dose calculation algorithm (Varian Medical Systems, Palo Alto, CA). Treatments were delivered on TrueBeam systems equipped with High-Definition or Millennium MLCs (2020–2024), as well as Halcyon and Ethos systems (2024) (Varian Medical Systems, Palo Alto, CA). PSQA findings were compared with high-risk failure modes identified by the ESTRO-AAPM task group [18]. Detection of these failure modes was used as a measure of PSQA program completeness.

**Fig. 2:**
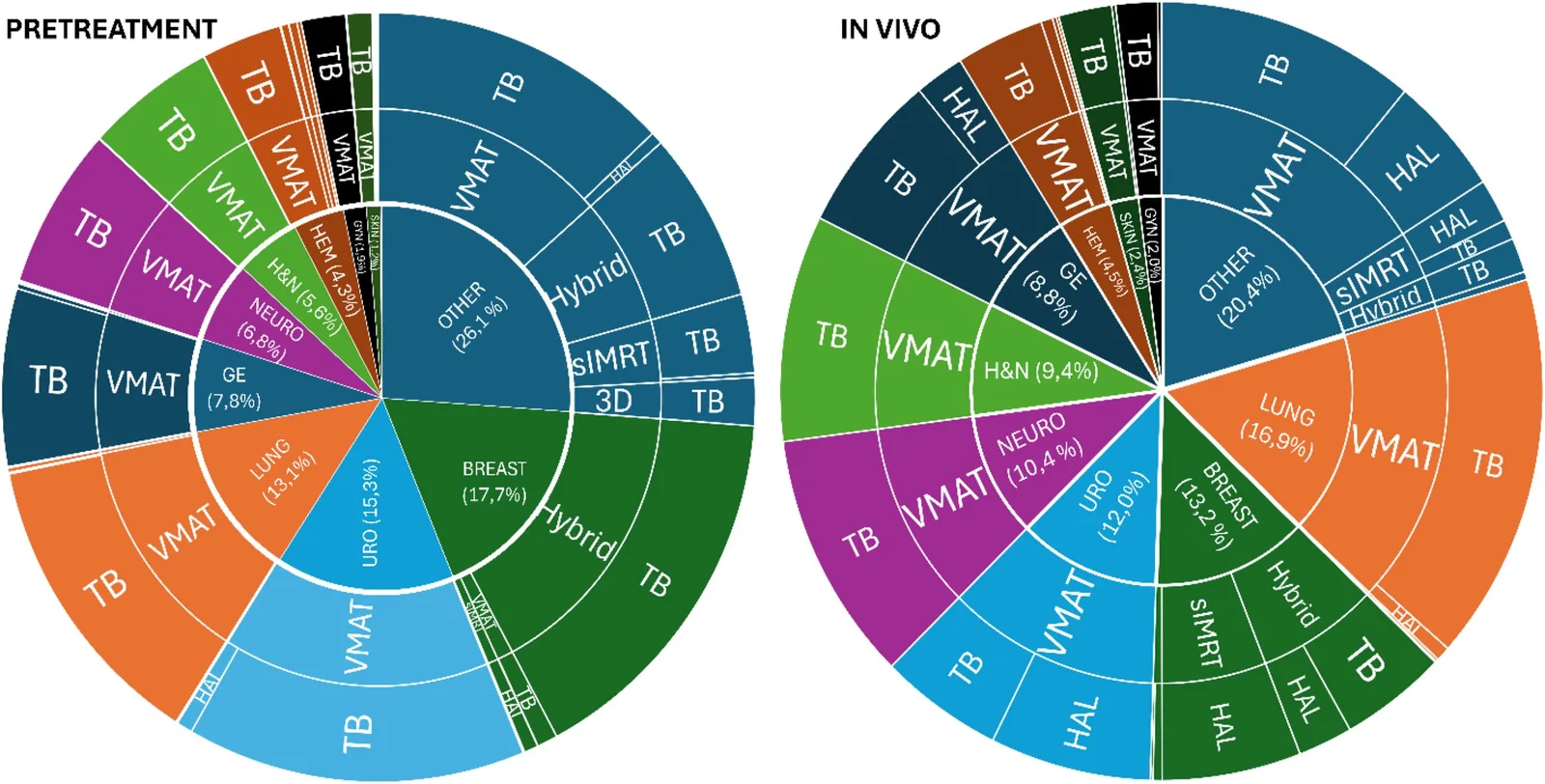
Illustration of the patient population evaluated using the PSQA program for pretreatment (5 years data) and *in vivo* plans (1 year data). Colors represent treatment indications (inner ring), treatment techniques used per treatment indication (second ring), treatment machines used per treatment indication (outer ring). Labels are removed from sections with low occurrence to improve readability. Abbreviations; TB: TrueBeam; HAL: Halcyon; VMAT: Volumetric Arc Therapy; sIMRT: Static Intensity Modulated Arc Therapy; 3D: 3D conformal therapy; URO: Urology; NEURO: Neurology; H&N: Head and Neck; GE: Gastroenterology; HEM: Hematology; GYN: Gynecology.

Program efficacy of the program was assessed using positive predictive value (PPV) calculated for each individual PSQA method individually and for combined workflow. The PPV is calculated as the percentage of true positive results relative to the total number of failed results. Failure was classified into four predefined failure categories: treatment plan generation, plan transfer and deliverability, inter- and intrafractional dose delivery. A PSQA failure attributed to the category under evaluation was considered a true positive; all others were classified as false positive.

Finally, PSQA outcomes were linked to potential improvements in treatment planning and delivery techniques by identifying recurring deviation patterns that could initiate protocol adaptations, planning strategies, and technological optimizations.

## 3 Results

Overall, 1.29% of treatment plans (*N* = 239) failed SDC evaluation (Fig. 3). False-positive results occurred in 0.55% of plans (*N* = 102) due to PSQA framework limitations, including limited portal dose image field of view (FOV), absence of electron contamination in the secondary dose algorithm and differences in HU binning discrepancies between the planning CT and CBCT. AAA-related dose calculation inaccuracies accounted for 0.39% (*N* = 72) of the plans, comprising dose overestimation in bone structures (0.32%, *N* = 59) and underestimation in lung tissue (0.07%, *N* = 13). Omission of immobilization devices during planning affected 0.21% of the plans (*N* = 39) while Acuros systematically underestimated dose for jaw-tracked fields smaller than 3 × 3 cm^2^ in 0.14% of plans (*N* = 26).

**Fig. 3:**
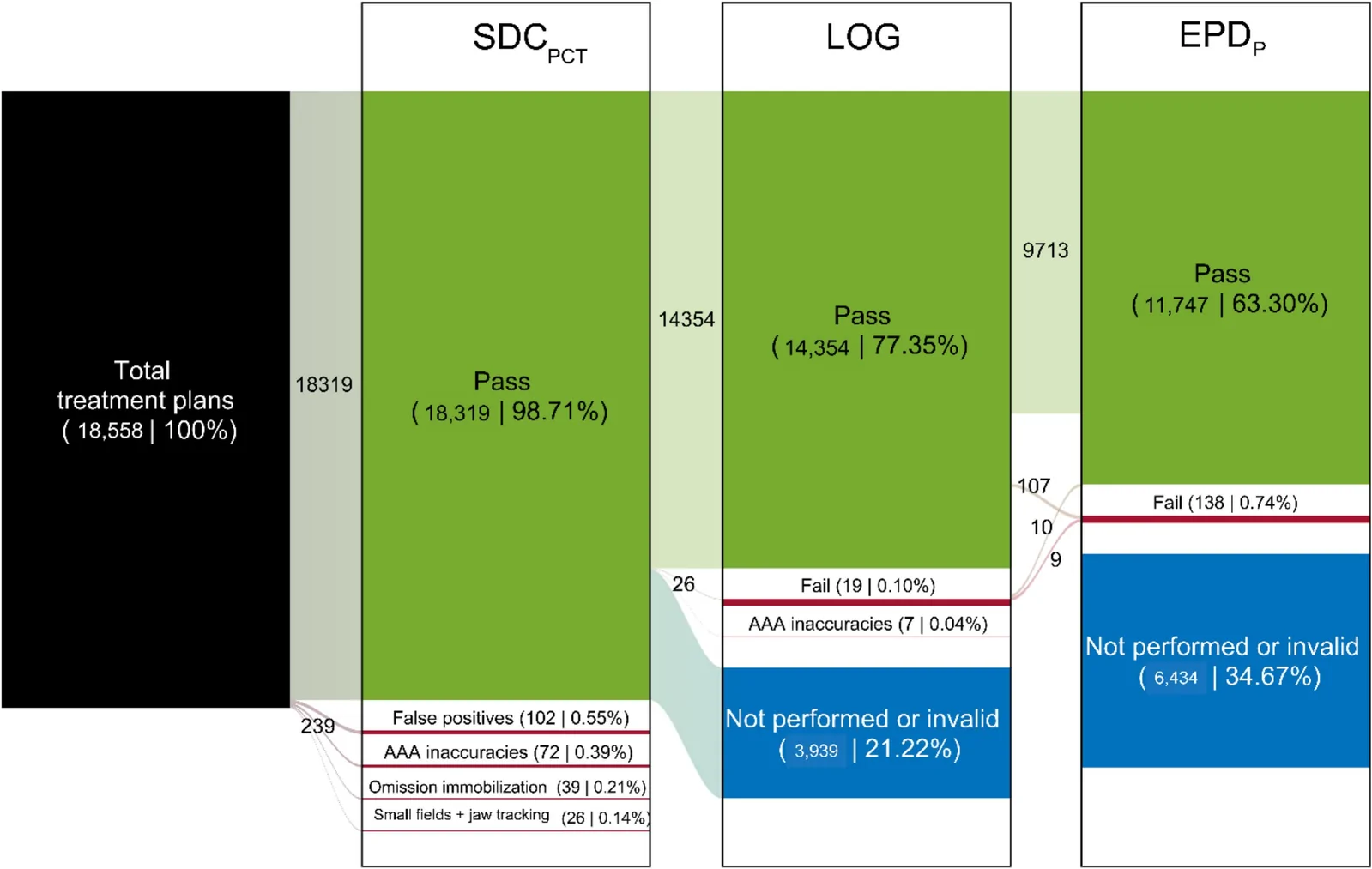
Overview of pretreatment plan validation results and failure modes (SDC_PCT_: Secondary Dose Calculation on the planning CT scan; LOG: dose verification based on machine log files; EPD_P_: pretreatment Electronic Portal Dosimetry). All percentages are stated relative to the total amount of treatment plans in the pretreatment workflow. Sum of total amount of LOG or the sum of total amount of EPD_P_ measurements corresponds to amount of passed SDC_PCT_ measurements, since no LOG of EPD_P_ is performed for plans with failed SDC_PCT_.

Log file analysis identified dose delivery discrepancies in 26 of 14,380 evaluated plans. Most failed LOG reports coincided with passed SDC results showing low agreement scores, suggesting dose calculation uncertainties, 7 of these plans were calculated with AAA. Only 9 failed LOG plans also failed the EPD_P_ PSQA, combining both combined dose calculation and dose delivery uncertainties. An additional 107 plans had a failed EPD_P_ result, typically involving heterogeneous and complex fluence patterns. In the Supplementary Materials (S1) an example of a case failing both LOG and EPD_P_ is provided. No protocol based PSQA was performed for 21.2% of LOG evaluations and for 34.7% of EPD_P_ according to protocol (*e.g.* palliation or phantom QA).

SDC_CBCT_ identified no clinically relevant deviations in 78.8% of treatment plans (Fig. 4). At least one failing fraction was observed in 21.2% of plans (*N* = 385), with patient alignment errors being the most common failure mode (9.1%, *N* = 166), predominantly in breast treatments. Additional alignment-related deviations were observed for lung treatments (*N* = 28), extremities (*N* = 21), bolus placement (*N* = 17), and shoulder positioning (*N* = 12). CBCT imaging limitations generated false positives in 5.1% of cases, primarily due to limited FOV (*N* = 53), HU differences between planning CT and CBCT (*N* = 31), and prosthesis-related artefacts (*N* = 8). The third category encompassed dose differences due to interfractional anatomical changes which were further subdivided into systematic and random differences. Systematic anatomical changes were observed in 3.9% of plans and included atelectasis or tumor shrinkage (*N* = 51), weight fluctuations (*N* = 11) and breast swelling or seroma formation (*N* = 9). Random anatomical variations occurred in 2.9% of plans and were mainly associated with diaphragm motion (*N* = 33) and variable abdominal air pockets (*N* = 20). Procedural deviations, such as excessive 4D-CT belt compression or omission of rectal balloons, were observed infrequently. Intrafractional motion affected only 0.5% of plans (*N* = 9), occurring in breast breath-hold treatments (*N* = 3), head-and-neck treatments with head displacements (*N* = 3), and abdominal treatments with variable air content (*N* = 3).

**Fig. 4:**
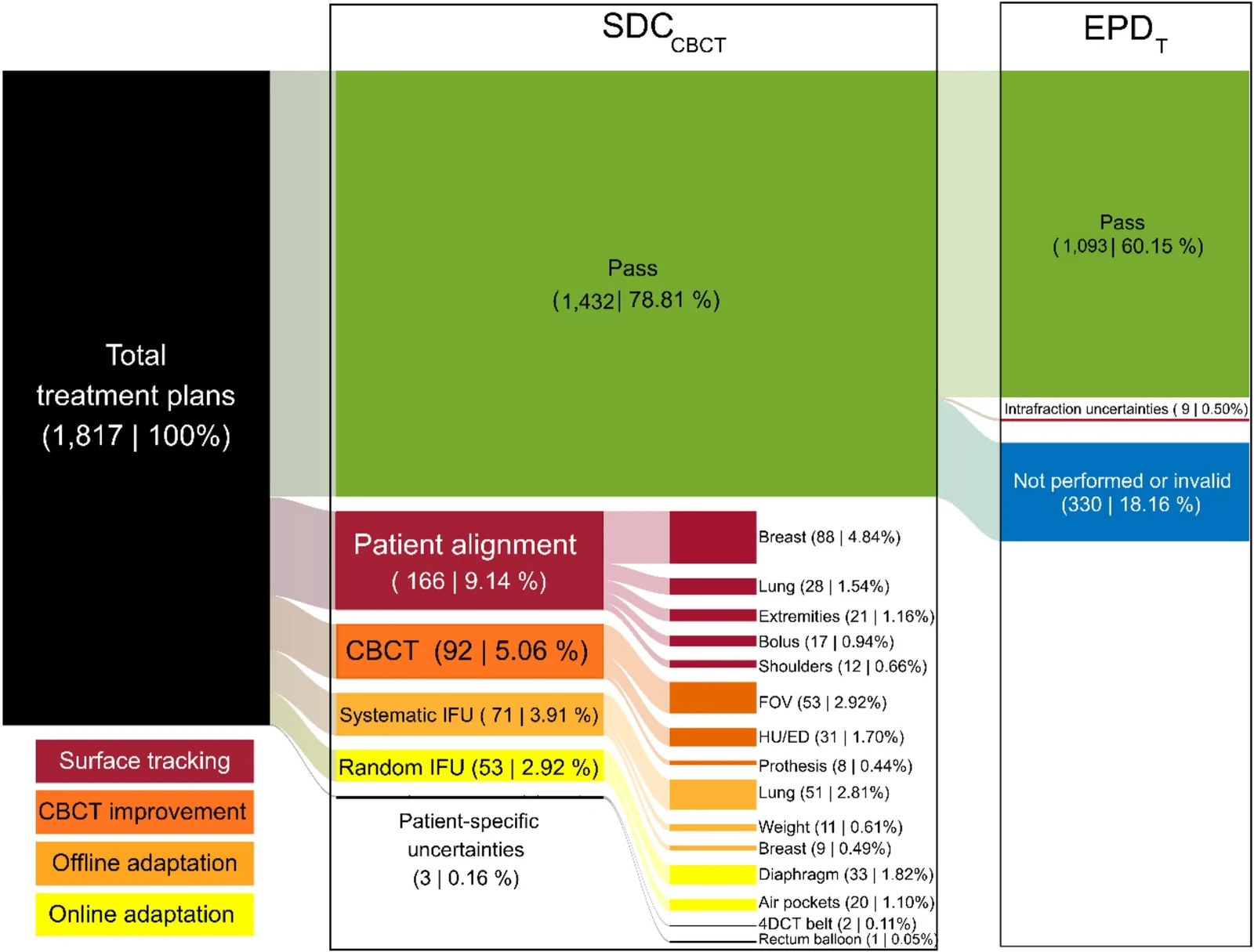
Overview of *in vivo* treatment dose verification results and failure modes (SDC_CBCT_: Secondary Dose Calculation on a CBCT scan; EPD_T_: *in vivo* Electronic Portal Dosimetry). All percentages are stated as relative to the total amount of treatment plans in the *in vivo* workflow. Sum of total amount of EPD_T_ corresponds to amount of passed SDC_CBCT_ measurements since no EPD_T_ is performed for plans with failed SDC_CBCT_. Abbreviations; IFU: Interfraction uncertainties.

Highly ranked ESTRO–AAPM failure modes were detected across all PSQA modalities (Table 1 and Table S1). PPV were generally low for individual methods (Table 2). SDC_PCT_ and SDC_CBCT_ achieved PPVs of 57% and 76%, respectively, matching the overall program because no false positives from unrelated failure modes occurred. EPD_T_ showed lower PPVs (1–62%) across failure mode categories. Combining PSQA methods consistently yielded equal or higher PPVs than any individual method. Potential strategies to improve future *in vivo* QA outcomes are summarized in Fig. 4.

**Table 1:** Summary of PSQA results for highly ranked failure modes by the ESTRO-AAPM task group based on RPN number. The amount of treatment plans averaged over one year is stated, and percentage is relative to the total treatment plans analyzed. Abbreviations; RPN: Risk Priority Number | SDC_PCT_: Secondary Dose Calculation on the planning CT scan; LO*G: Log-file based QA; EPID*_*P*_*: pretreatment Electronic Portal Dosimetry; SDC*_*CBCT*_*: Secondary Dose Calculation on a CBCT scan; EPD*_*T*_*: in vivo* Electronic Portal Dosimetry; TPS: Treatment Planning System; MLC: Multi Leaf Collimator; DLG: Dosimetric Leaf Gap; OF: Output Factor; PDD: Percentage Depth Dose; TMR: Tissue Maximum Ratio; OAF: Off-Axis Factor; MU: Monitor Unit; HU: Hounsfield Unit; FFF: Flattening Filter Free.

**Failure mode ESTRO-AAPM FMEA: Top ranked based on risk**		**Failure mode found**	**SDCPCT**	**LOG**	**EPDP**	**SDCCBCT**	**EPDT**	**SUM**
TPS algorithm limitation		AAA inaccuracies	14 | 0.39%	NP	NP	NF	NP	27 | 0.74%
		Omission immobilization	8 | 0.21%	NP	NP	NF	NP	
		Small fields + jaw tracking	5 | 0.14%	NP	NP	NF	NP	
MLC (DLF or transmission) / OF/PDD/TMR/OAF modeling errors	Error during measurement	Not found	NF	NF	0	NF	0	0
	Error during commissioning	Not found	NF	NF	0	NF	0	0
cGy/MU incorrect	Output miscalibration		NF	NF	NF	NF	NF	NF
	Hardware error	Not found	NF	NF	0	NF	0	0
Linac isocenter incorrect	Error coincidence imaging, mechanical isocenter or treatment isocenter	Not found	NF	NF	NF	NF	0	0
	Error in ODI, imaging or lasers	Not found	NF	NF	NF	0	0	0
External patient position variation		Patient alignment	NF	NF	NF	166 | 9.1%		178 | 9.8%
		Patient specific issues	NF	NF	NF	3 | 0.2%		
		Intrafraction motion	NF	NF	NF	NF	9 | 0,5%	
Patient weight variation		Weight-loss or -gain	NF	NF	NF	11 | 0.6%*		11 | 0.6%*
Challenging plan parameters		Overmodulation	NF	30 | 0.80%		NF	NP	30 | 0.80%
Error in CT density to HU conversion		HU/ED difference CBCT/PCT	NF	NF	NF	92 | 1.7%		31 | 1.7%
Internal organ density or position variation		Systematic interfraction errors	NF	NF	NF	71 | 3.9%		124 | 6.8%
		Random interfraction errors	NF	NF	NF	53 | 2.9%		
*Patient weight variation not included in sum. These values are already accounted for in failure mode systematic interfraction errors. NF=Not found, NP=Not performed **SUM**			27 | 0.74%	30 | 0.80%		333 | 18.33%		390 | 19.87%

**Table 2:** Overview of positive predictive values for 4 different failure categories per PSQA method. SDC_PCT_: Secondary Dose Calculation on the planning CT scan; LOG: Logfile-based QA; EPID_P_: pretreatment Electronic Portal Dosimetry; SDC_CBCT_: Secondary Dose Calculation on a CBCT scan; EPD_T_: *in vivo* Electronic Portal Dosimetry; NA = Not applicable.

Positive predictive values (%)	SDC_PCT_	LOG	EPD_P_	SDC_CBCT_	EPD_T_	PSQA program
Treatment plan generation	57	54	36	NA	6	57
Plan transfer and deliverability	NA	7	37	NA	1	59
Interfractional dose delivery	NA	NA	NA	76	62	76
Intrafractional dose delivery	NA	NA	NA	NA	2	17

## 4 Discussion

This large-scale retrospective study showed that combining SDC, log file analysis, EPD, and *in vivo* dosimetry enables detection of complementary failure modes throughout the radiotherapy workflow. *In vivo* PSQA identified the most clinically relevant deviations, while pretreatment methods were most effective for planning and deliverability issues. Despite limited PPVs of individual methods, their combination improved error detection and interpretation, supporting the use of integrated PSQA frameworks to enhance radiotherapy quality and safety.

The PSQA program identified failure modes across the entire radiotherapy workflow. Consistent with the ESTRO-AAPM task group ranking, limitations of TPS dose calculation algorithms were among the most important findings. The observed dose overestimation in bone and underestimation in lung tissue by AAA agrees with previous reports [19]. In addition, a small subset of plans showed systematic dose underestimation when jaw tracking was applied to small fields, confirming earlier findings by Swinnen et al. [20]. Despite internal guidelines discouraging jaw tracking for very small fields, PSQA identified plans that did not adhere to this recommendation. Furthermore, 0.21% of plans failed due to omission of immobilization devices during treatment planning. Inclusion of these devices in the SDC_PCT_ evaluation [21] revealed clinically relevant dose discrepancies, resulting in a protocol change requiring their consideration during treatment planning.

Another failure mode involved inaccuracies in TPS input data, including multi leaf collimator (MLC) modeling, dynamic leaf gap (DLG) values, MLC transmission values, percentage depth dose (PDD) curves, tissue-maximum-ratio (TMR), off-axis factors (OAF), *etc.* A distinction was made between errors arising from inconsistencies during measurement of these data and errors introduced during TPS commissioning. PDIs were generated from the same measurement data used by the TPS. This caused the PSQA methods SDC_P_, LOG and SDC_CBCT_ to miss errors in measurement of the TPS data. Since the EPD methods, both pretreatment and *in vivo*, made use of an independent fluence measurement, these PSQA methods could flag this failure mode. This reinforces the need for accurate acquisition of TPS input data and for PSQA methods that are fully independent of TPS commissioning. As noted by Wong et al., PSQA effectiveness diminishes when both systems rely on the same underlying data [22]. An improvement to the framework could be the adoption of a direct 3D forward dose calculation instead of using predicted 2D PDIs for the SDC and LOG PSQA methods, using independently measured input data. The use of standardized “golden beam data” rather than site-specific commissioning measurements may further reduce variability and enhance reliability [23]. The failure mode that could not be found by the PSQA framework is the output calibration. Since a daily calibration of our EPD panel neglecting linac output variations is performed, the impact of output variations on the portal dose measurements is removed (< 1%). This failure mode in return could be found by performing regular machine output measurements. Thus, combining a PSQA program with rigorous machine QA can be crucial to ensure a complete and robust PSQA program.

A severe failure mode can be a mismatch between planned and delivered MLC positions. By combining LOG PSQA with EPID measurements, a distinction could be made between plan transfer errors, incomplete EPD acquisitions, and deliverability issues *e.g.* overmodulated MLC sequences. EPD and LOG PSQA continue to be valuable tools when implementing new treatment techniques and evaluating emerging platforms. Additionally, correlating EPD PSQA outcomes with plan complexity metrics may offer predictive insight into deliverability during treatment planning, an avenue for future research [24], [25], [26]. Other high severity failure modes (including incorrect energy selection, partial delivery, or incorrect MU) could also be detected through EPD and/or LOG PSQA. Although LOG PSQA offers a less labor-intensive alternative to measurement based PSQA, not all delivery issues can be captured by this method alone. Uncertainties such as erroneous beam tuning and slowly changing beam properties can only be found with an independent fluence measurement.

The most frequently observed failure modes were identified by the *in vivo* PSQA methods. Esposito et al. reported out-of-tolerance rates for EPID-based *in vivo* dosimetry of 5–18% depending on technique and system, which is consistent with the 21.2% of plans showing at least one failed SDC_CBCT_ in the cohort [27]. *In vivo* PSQA provided valuable insight into how interfractional and intrafractional variations influence the delivered dose. Although most finding are reported in literature [27], [28], [29], the *in vivo* PSQA results offer an efficient way for detecting and mitigating such uncertainties during the treatment. The incidence of positioning-related deviations (~9%) and anatomical changes (~3–7%) observed in the cohort is consistent with previously reported *in vivo* dosimetry studies, where positioning errors were reduced from approximately 10% to 3–5% and anatomical variation from ~10% to ~3–4% following workflow adaptations [30]. Despite established clinical protocols, patient-specific errors (such as the omission of a rectal balloon during treatment) can still occur and may substantially affect treatment outcome. The EPD_T_ findings highlight the limited, yet clinically relevant role of intrafractional motion monitoring, particularly in anatomically mobile regions or motion-sensitive protocols. As these results are evaluated only after treatment delivery, their immediate clinical actionability is more limited for hypofractionated treatments than for hyperfractionated treatments. Real-time evaluation would be required to enable adaptive intervention during treatment, but can give insight for protocol improvements.

The PPV provided insight into the reliability of a positive PSQA result. As shown in Table 2, the PPVs of the individual PSQA methods across the different failure mode categories were generally very low. This is primarily explained by the low prevalence of true failures: only a small subset of plans exhibited failing PSQA results. The low prevalence reflects the improvements in treatment delivery techniques driven by insights from the PSQA program. For the treatment plan generation and interfractional dose delivery categories, the PPVs are equal to those of the overall program, as false positives from other failure modes cannot occur. In the case of SDC_CBCT_, each dose was compared to a SDC_PCT_, thereby minimizing differences related to three-dimensional dose calculation algorithms. In contrast, substantial increases in PPV are observed between plan transfer and deliverability, and intrafractional dose delivery. Both categories are characterized by a low prevalence, meaning that most detected failures are attributable to other failure modes rather than the one being tested. Although EPD_T_ can detect all failure categories, its PPVs remain low, as the results may reflect multiple underlying error sources. Consequently, interpretation is challenging when this method is used in isolation, since dose differences arising from different failure modes may compensate for each other and obscure the true cause. The higher PPV observed for the PSQA program demonstrated that combining multiple methods enhances the robustness of error detection and improved the interpretability of the results. PPVs can additionally be increased by improving PSQA methods and hence reducing the number of false positives. *E.g.* using more accurate Monte Carlo algorithms and Hypersight CBCT technology with extended FOV can further enable reliable dose evaluation [31]. Artificial intelligence may strengthen future PSQA frameworks by enabling deeper analysis of complex datasets, improving failure mode identification and supporting clinical decision making [32], [33], [34]. The PPV should be interpreted in the context of the applied gamma criteria and pass rate thresholds. Stricter thresholds typically increase sensitivity but may lead to more false positives and a lower PPV, whereas less stringent thresholds can improve PPV at the cost of increased false negatives.

Evaluating PSQA results allow for identifying limitations within the radiotherapy workflow but also in supporting the safe implementation and evaluation of new technologies in clinical practice [28]. For example, surface tracking may improve setup accuracy in breast and lung treatments when arm positioning often leads to dose discrepancies [35], [36]. Offline adaptation may be appropriate for interfractional changes such as tumor regression or weight loss [29], whereas online adaptation may be required for intrafractional variations such as air pocket movement [37], [38]. Within the current framework, treatment plans are primarily flagged based on gamma analysis. While the standard (3%, 3 mm) criteria were applied, tailoring gamma criteria per treatment indication, such as (5%, 1 mm) for stereotactic treatments, may provide additional value. The gamma metric is sensitive to dose discrepancies but not always linked directly to clinical relevance. The integration of more clinically meaningful endpoints, such as dose–volume histogram parameters or normal tissue complication probability metrics, could enhance decision making. In addition, dose accumulation approaches may provide a more complete understanding of the impact of interfractional variations. To correctly evaluate the impact of PSQA results, good registration of adaptation rates and reasons for the adaptation should be tracked.

This large-scale retrospective analysis demonstrates that complementary pretreatment and *in vivo* PSQA methods detect different classes of treatment deviations and provide distinct but valuable information. Maintaining methodological independence while integrating these approaches within a coordinated framework improves the interpretation of QA findings, facilitates the identification of underlying causes, and supports targeted corrective actions. Together, these results underscore the importance of a comprehensive PSQA strategy for optimizing patient treatments and ensuring robust quality assurance in modern radiotherapy.

## CRediT authorship contribution statement

**Esther Decabooter:** Writing – review & editing, Writing – original draft, Methodology, Formal analysis, Conceptualization. **Sebastiaan M.J.J.G. Nijsten:** Writing – review & editing, Methodology, Conceptualization.

## Ethics declaration

### Informed consent and patient details

The authors have NOT obtained informed consent from participants or their legal representatives. Only QA results are evaluated, not patient specific data.

### Studies in Human

This study was performed in compliance with relevant laws, regulatory frameworks and guidelines where the research took place. This study was approved by the Internal Review Board. (Approval No. PO0593)

## Declaration of competing interest

The authors declare that they have no known competing financial interests or personal relationships that could have appeared to influence the work reported in this paper.

## References

[1] Fiagan Y.A.C., N‘Guessan K.J.F., Diakité A., Adjenou K.V., Gevaert T., Verellen D. (2025). Evaluation of using an Octavius 4D measuring system for patient-specific VMAT quality assurance. Radiation (Basel).

[2] Khaophong J., Yongvithisatid P., Pairat K., Dechsupa P., Tangboonduangjit P., Asavaphatiboon S. (2019). Evaluation of Delta 4 system in patient specific QA for VMAT technique: retrospective lung VMAT cases. J Phys Conf Ser.

[3] Urso P., Lorusso R., Marzoli L., Corletto D., Imperiale P., Pepe A. (2018). Practical application of Octavius® -4D: characteristics and criticalities for IMRT and VMAT verification. J Applied Clin Med Phys.

[4] Chaswal V., Weldon M., Gupta N., Chakravarti A., Rong Y. (2014). Commissioning and comprehensive evaluation of the ArcCHECK cylindrical diode array for VMAT pretreatment delivery QA. J Applied Clin Med Phys.

[5] Niroomand-Rad A., Chiu-Tsao S., Grams M.P., Lewis D.F., Soares C.G., Van Battum L.J. (2020). Report of AAPM task group 235 Radiochromic film dosimetry: an update to TG-55. Med Phys.

[6] Borca V.C., Pasquino M., Russo G., Grosso P., Cante D., Sciacero P. (2013). Dosimetric characterization and use of GAFCHROMIC EBT3 film for IMRT dose verification. J Applied Clin Med Phys.

[7] Howard M.E., Herman M.G., Grams M.P. (2020). Methodology for radiochromic film analysis using FilmQA pro and ImageJ. PLoS One.

[8] Wendling M., Louwe R.J.W., McDermott L.N., Sonke J., Van Herk M., Mijnheer B.J. (2006). Accurate two-dimensional IMRT verification using a back-projection EPID dosimetry method. Med Phys.

[9] McDermott L.N., Wendling M., Sonke J.-J., Van Herk M., Mijnheer B.J. (2007). Replacing pretreatment verification with in vivo EPID dosimetry for prostate IMRT. Int J Radiat Oncol Biol Phys.

[10] Pasma K.L., Kroonwijk M., Quint S., Visser A.G., Heijmen B.J.M. (1999). Transit dosimetry with an electronic portal imaging device (EPID) for 115 prostate cancer patients. Int J Radiat Oncol Biol Phys.

[11] Vieira S.C., Dirkx M.L.P., Pasma K.L., Heijmen B.J.M. (2002). Fast and accurate leaf verification for dynamic multileaf collimation using an electronic portal imaging device. Med Phys.

[12] Van Elmpt W., McDermott L., Nijsten S., Wendling M., Lambin P., Mijnheer B. (2008). A literature review of electronic portal imaging for radiotherapy dosimetry. Radiother Oncol.

[13] Essers M., Lanson J.H., Mijnheer B.J. (1993). In vivo dosimetry during conformal therapy of prostatic cancer. Radiother Oncol.

[14] Essers M., Mijnheer B. (1999). In vivo dosimetry during external photon beam radiotherapy. Int J Radiat Oncol Biol Phys.

[15] Şahin L., Tunçel N. (2025). Retrospective evaluation of patient-specific intensity-modulated radiotherapy plan verification data in a single department. Int J Radiol Radiat Ther.

[16] Osei E., Graves S., Darko J. (2022). Retrospective analysis of portal dosimetry pre-treatment quality assurance of intracranial SRS/SRT VMAT treatment plans. J Radiother Pract.

[17] Decabooter E., Essers M., Gershkevitsh E., Hussein M., Kry S.F., Lehmann J. (2026). Patient specific quality assurance: the final safety net in radiotherapy. Radiother Oncol.

[18] O'Daniel J., Hernandez V., Clark C., Esposito M., Lehmann J., McNiven A. (2025). Which failures do patient-specific quality assurance systems need to catch?. Med Phys.

[19] Robinson D. (2008). Inhomogeneity correction and the analytic anisotropic algorithm. J Applied Clin Med Phys.

[20] Swinnen A.C.C., Öllers M.C., Roijen E., Nijsten S.M., Verhaegen F. (2017). Influence of the jaw tracking technique on the dose calculation accuracy of small field VMAT plans. J Applied Clin Med Phys.

[21] Oulhouq Y., Rrhioua A., Bakari D., Zerfaoui M., Krim D. (2022). The effect of carbon fibre treatment couch with and without immobilisation devices on radiotherapy dose calculation using three different planning algorithms and photon beam energies. J Radiother Pract.

[22] Wong L.M., Pawlicki T. (2024). A systems theory-based safety assessment of pre-treatment patient-specific quality assurance for intensity-modulated treatments in a single-vendor environment. Radiother Oncol.

[23] Yousif Y.A.M., Gastaldo J., Baldock C. (2022). Golden beam data provided by linear accelerator manufacturers should be used in the commissioning of treatment planning systems. Australas Phys Eng Sci Med.

[24] Hernandez V., Hansen C.R., Widesott L., Bäck A., Canters R., Fusella M. (2020). What is plan quality in radiotherapy? The importance of evaluating dose metrics, complexity, and robustness of treatment plans. Radiother Oncol.

[25] Chen L., Luo H., Li S., Tan X., Feng B., Yang X. (2023). Pretreatment patient-specific quality assurance prediction based on 1D complexity metrics and 3D planning dose: classification, gamma passing rates, and DVH metrics. Radiother Oncol.

[26] Hernandez V., Saez J., Pasler M., Jurado-Bruggeman D., Jornet N. (2018). Comparison of complexity metrics for multi-institutional evaluations of treatment plans in radiotherapy. Phys Imaging Radiat Oncol.

[27] Esposito M., Piermattei A., Bresciani S., Orlandini L.C., Falco M.D., Giancaterino S. (2021). Improving dose delivery accuracy with EPID in vivo dosimetry: results from a multicenter study. Strahlenther Onkol.

[28] Bossuyt E., Weytjens R., Nevens D., De Vos S., Verellen D. (2020). Evaluation of automated pre-treatment and transit in-vivo dosimetry in radiotherapy using empirically determined parameters. Phys Imaging Radiat Oncol.

[29] Hattu D., Taasti V.T., Emans D., Velders M., Van Der Stoep J., Vullings A. (2025). Effects of anatomical changes on dose and normal tissue complication probabilities and the benefit of replanning in photon and proton therapy of lung cancer patients. Radiotherapy and Oncology Radiother Onco.

[30] Bossuyt E., Nevens D., Weytjens R., Taieb Mokaddem A., Verellen D. (2023). Assessing the impact of adaptations to the clinical workflow in radiotherapy using transit in vivo dosimetry. Phys Imaging Radiat Oncol.

[31] Bogowicz M., Lustermans D., Taasti V.T., Hazelaar C., Verhaegen F., Fonseca G.P. (2024). Evaluation of a cone-beam computed tomography system calibrated for accurate radiotherapy dose calculation. Phys Imaging Radiat Oncol.

[32] Heising L.M., Verhaegen F., Scheib S.G., Jacobs M.J.G., Ou C.X.J., Mottarella V. (2025). Toward a human-centric co-design methodology for AI detection of differences between planned and delivered dose in radiotherapy. J Applied Clin Med Phys.

[33] Wolfs C.J.A., Canters R.A.M., Verhaegen F. (2020). Identification of treatment error types for lung cancer patients using convolutional neural networks and EPID dosimetry. Radiother Oncol.

[34] Van Den Berg K., Wolfs C.J.A., Verhaegen F. (2024). A 3D transfer learning approach for identifying multiple simultaneous errors during radiotherapy. Phys Med Biol.

[35] Freislederer P., Batista V., Öllers M., Buschmann M., Steiner E., Kügele M. (2022). ESTRO-ACROP guideline on surface guided radiation therapy. Radiother Oncol.

[36] Freislederer P., Kügele M., Öllers M., Swinnen A., Sauer T.-O., Bert C. (2020). Recent advances in surface guided radiation therapy. Radiat Oncol.

[37] Lu L., Zhang Z., Qi P. (2024). A review of online adaptive radiation therapy. Appl Radiat Oncol.

[38] Liu H., Schaal D., Curry H., Clark R., Magliari A., Kupelian P. (2023). Review of cone beam computed tomography based online adaptive radiotherapy: current trend and future direction. Radiat Oncol.

