# Supplementary material for: Evidence-based design of a patient-specific quality assurance program through retrospective analysis of pretreatment and in vivo results
Source: Phys Imaging Radiat Oncol. 2026 Oct 9;41:101106. doi: 10.1016/j.phro.2026.101106 (PMC13682176; doi:10.1016/j.phro.2026.101106)
Supplement: Supplementary file 1 [file mmc1.pdf]

Supplementary Materials

A. Technical implementation of the PSQA program

All PSQA methods are based on a two-dimensional portal dose image (PDI), either predicted or measured, which is used to reconstruct a three-dimensional dose distribution on a CT data set [1]. Predicted PDIs were generated using a model-based approach [2], while measured PDIs were derived from MV portal images using a dose conversion model [3]. Three-dimensional dose reconstruction was performed using the XVMC Monte Carlo dose calculation engine [4].

Flattening filter free (FFF) beams on TrueBeam linacs were excluded from the analysis because no portal dose models were commissioned for these energies. To compensate for long-term detector response variations, a daily absolute EPID calibration was performed.

Dose comparisons were evaluated using a global 3D gamma analysis with criteria of 3%/3 mm. Gamma analysis was restricted to the high-dose region, defined as voxels receiving at least 50% of the prescribed dose. A result was classified as failed when the gamma pass rate was below 95%. The 3%/3 mm criterion was selected because it remains the clinical standard for PSQA and is retained as a benchmark within AAPM TG-218 [5].

PSQA results, together with treatment plan metadata, were stored in a centralized Microsoft SQL database and extracted using Microsoft Power BI for retrospective analysis.

For the *in vivo* workflow, each SDC_{CBCT} reconstruction was compared with the corresponding SDC_{PCT} dose distribution to reduce differences originating from the used 3D dose calculation algorithm. Transit portal dosimetry results (EPD_T) were normalized to the reference dose using the maximum histogram counts. This relative dosimetry approach compensates for residual uncertainties in the transit dose conversion model while maintaining sensitivity to clinically relevant failure modes.

[1] Van Elmpt WJC, Nijsten SMJJG, Schiffeleers RFH, Dekker ALAJ, Mijnheer BJ, Lambin P, et al. A Monte Carlo based three-dimensional dose reconstruction method derived from portal dose images. Med Phys 2006;33:2426–34. https://doi.org/10.1118/1.2207315.

[2] Baeza JA, Wolfs CJA, Nijsten SMJJG, Verhaegen F. Validation and uncertainty analysis of a pre-treatment 2D dose prediction model. Phys Med Biol 2018;63:035033. https://doi.org/10.1088/1361-6560/aa9d0c.

[3] Nijsten SMJJG, Van Elmpt WJC, Jacobs M, Mijnheer BJ, Dekker ALAJ, Lambin P, et al. A global calibration model for EPIDs used for transit dosimetry. Med Phys 2007;34:3872–84. https://doi.org/10.1118/1.2776244.

[4] Fippel M. Fast Monte Carlo dose calculation for photon beams based on the VMC electron algorithm. Med Phys 1999;26:1466–75. https://doi.org/10.1118/1.598676.

[5] Miften M, Olch A, Mihailidis D, Moran J, Pawlicki T, Molineu A, et al. Tolerance limits and methodologies for IMRT measurement-based verification QA : *Recommendations of AAPM Task Group No. 218* . Med Phys 2018;45. https://doi.org/10.1002/mp.12810.

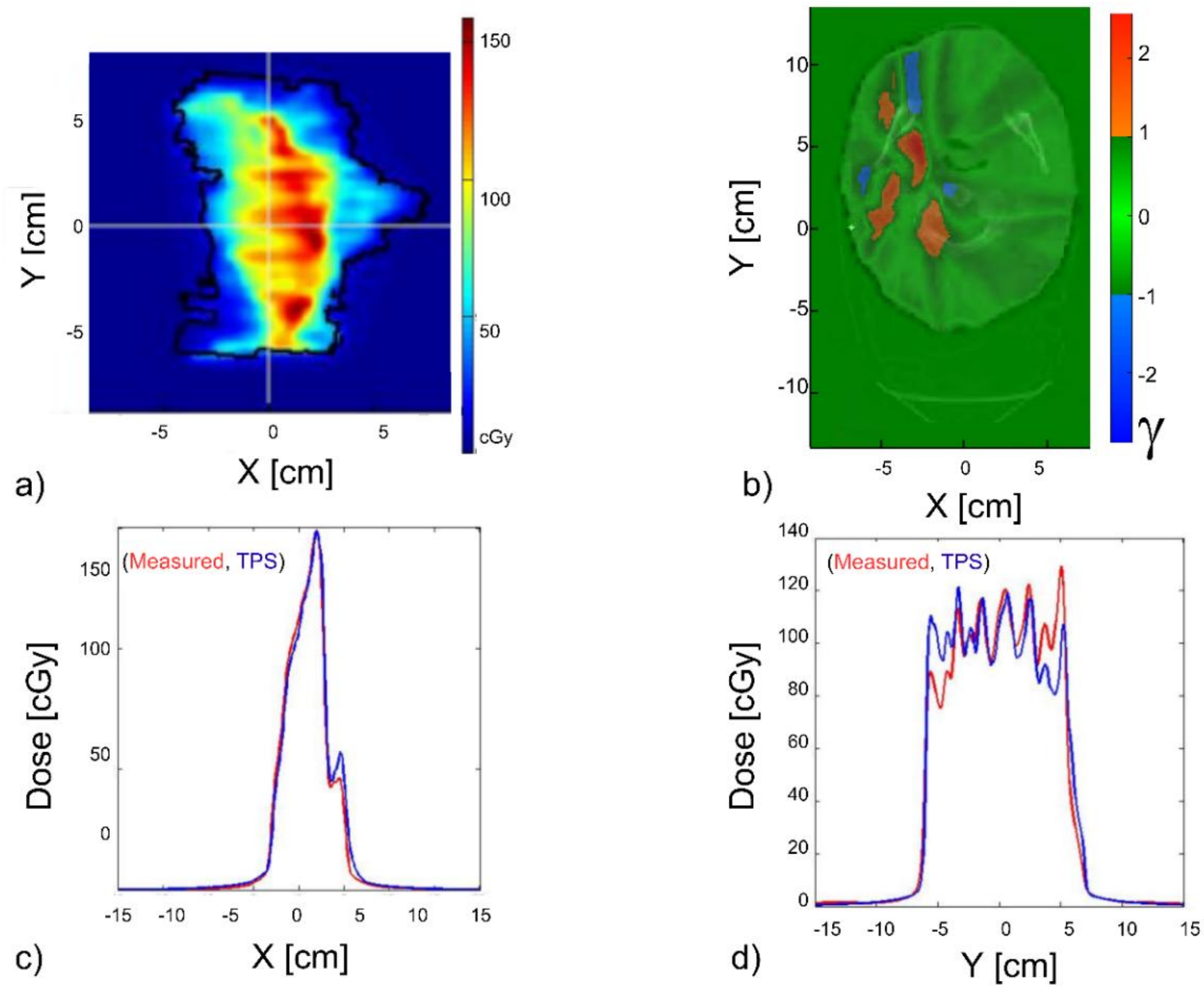

Figure S1: Results of EPD based pre-treatment PSQA in case of an overmodulated treatment plan showing (a) the measured 2D portal dose of a treatment field, (b) a slice of the 3D gamma comparison between the TPS dose and the EPD measurement (red: a higher dose is measured; blue: a lower dose is measured), and (c,d) crossline and inline dose profiles through the center of the projected isocenter in the EPID plane (red: measured dose; blue: TPS dose).

Table S1. Summary of PSQA results for highly ranked failure modes by the ESTRO-AAPM task group based on severity. The amount of treatment plans averaged over one year is stated, and the percentage is relative to the total treatment plans analyzed. Abbreviations; RPN: Risk Priority Number | SDC_{PCT}: Secondary Dose Calculation on the planning CT scan; LOG: Log file based QA; $EPID_{P}$: pre-treatment Electronic Portal Dosimetry; SDC_{CBCT}: Secondary Dose Calculation on a CBCT scan; EPD_{T}: *in vivo* Electronic Portal Dosimetry; TPS: Treatment Planning System; MLC: Multi Leaf Collimator; MU: Monitor Unit; FFF: Flattening Filter Free.

<table>
<tr><th colspan="2">Failure mode ESTRO-AAPM FMEA</th><th>Failure mode found</th><th>SDC_{PCT}</th><th>LOG</th><th>EPD_{P}</th><th>SDC_{CBCT}</th><th>EPD_{T}</th><th>SUM</th></tr>
<tr><td colspan="8">Top Ranked failure modes based on severity</td><td></td></tr>
<tr><td>MLC delivery sequence does not match planned</td><td></td><td>Overmodulation</td><td>NF</td><td colspan="2">30 | 0.80 %</td><td>NF</td><td>NP</td><td>30 | 0.80 %</td></tr>
<tr><td colspan="2">Collision</td><td>Not found</td><td>NF</td><td>NF</td><td>NF</td><td>NF</td><td>0</td><td>0</td></tr>
<tr><td>TPS isocenter incorrect</td><td>Error in laterality, isocenter location,coordinate system,…</td><td>Not found</td><td>NF</td><td>NF</td><td>NF</td><td>0</td><td>0</td><td>0</td></tr>
<tr><td colspan="2">Patient alignment incorrect</td><td>Patient alignment</td><td>NF</td><td>NF</td><td>NF</td><td colspan="2">166 | 9.1 %</td><td>166 | 9.1 %</td></tr>
<tr><td colspan="2">Energy/Modality error/FF vs FFF</td><td>Not found</td><td>NF</td><td>0</td><td>0</td><td>NF</td><td>0</td><td>0</td></tr>
<tr><td colspan="2">Partial or excess treatment delivery</td><td>Not found</td><td>NF</td><td>0</td><td>0</td><td>NF</td><td>0</td><td>0</td></tr>
<tr><td>MU incorrect</td><td>Due to human error</td><td>Not found</td><td>NF</td><td>0</td><td>0</td><td>NF</td><td>0</td><td>0</td></tr>
<tr><td>Wrong plan or patient</td><td>Data transfer error</td><td>Not found</td><td>NF</td><td>NF</td><td>NF</td><td>0</td><td>0</td><td>0</td></tr>
<tr><td colspan="3">SUM</td><td>-</td><td colspan="2">30 | 0.8 %</td><td colspan="2">166 | 9.1 %</td><td>196 | 9.9%</td></tr>
<tr><td colspan="9">*Patient weight variation not included in sum. These values are already accounted for in failure mode systematic interfraction errors.
NF=Not found, NP=Not performed</td></tr>
</table>
